# ESTIMATION OF H_P_(3) AMONG STAFF MEMBERS IN TWO NUCLEAR MEDICINE UNITS IN FINLAND

**DOI:** 10.1093/rpd/ncaa096

**Published:** 2020-07-14

**Authors:** C Lindholm, A Pekkarinen, O Sipilä, A-L Manninen, M Lehtinen, T Siiskonen

**Affiliations:** 1 STUK-Radiation and Nuclear Safety Authority, POB 14, Helsinki 00811, Finland; 2 HUS Medical Imaging Center, Helsinki University Hospital, Helsinki, Finland; 3 Department of Physics, University of Helsinki, Helsinki, Finland; 4 HUS Medical Imaging Center, Clinical Physiology and Nuclear Medicine, University of Helsinki and Helsinki University Hospital, Helsinki, Finland; 5 OYS Department of Nuclear Medicine and Radiology, Oulu University Hospital, Oulu, Finland; 6 Medical Research Center Oulu, Oulu University Hospital and University of Oulu, Oulu, Finland

## Abstract

The eye lens exposure among 16 technicians in two nuclear medicine departments at university hospitals in Finland was investigated by measuring the operational quantity H_p_(3) using EYE-D dosemeters. For all workers, the annual mean H_p_(3) was estimated to be 1.1 mSv (max. 3.9 mSv). The relation between H_p_(3) to routinely monitored personal dose equivalent H_p_(10) was clearly correlated. Considering individual dose measurement periods (2–4 weeks), the H_p_(3)/H_p_(10) ratio was 0.7 (Pearson’s coefficient *r* = 0.90, *p* < 0.001, variation of ratio 0.1–2.3). The variation decreased considerably with increasing H_p_(10) (*σ*^2^ = 0.04 vs. 0.43 for H_p_(10) > 0.1 mSv vs. < 0.1 mSv, respectively), i.e. higher H_p_(10) predicts H_p_(3) more reliably. Moreover, annual H_p_(10) data from national dose register during 2009–2018 were used to derive the annual H_p_(3) applying the H_p_(3)/H_p_(10) ratio. The data from Finnish nuclear medicine departments imply that routine measurements of H_p_(3) among nuclear medicine technicians are not justified.

## INTRODUCTION

The allowed annual equivalent dose to the lens of the eye for occupational exposure has been prominently reduced from 150 mSv to a mean value of 20 mSv obtained over a period of 5 years and with no single year exceeding 50 mSv. The transition is based on epidemiological studies showing that the eye lens is more sensitive to radiation than previously considered and that the threshold for eye lens opacities is much lower or that there may not be a threshold at all. Dose response in cataract formation was observed among atom bomb survivors investigated 55 years post exposure^(1)^. Examinations of lens opacities due to fractionated and protracted irradiation among Chernobyl cleanup workers 12 and 14 years after exposure suggested that there is an accumulated dose threshold of 0.35 Sv for certain types of cataract and opacity. Further, studies on radiological technologists^(2, 2)^ and interventional cardiologists^(4)^ implied that cataracts may occur after exposure to much lower doses than was earlier comprehended. Based on these, and a large number of other investigations, the International Commission on Radiological Protection (ICRP) concluded that the lifetime dose threshold for induction of cataracts needs to be lowered to an absorbed dose of 0.5 Gy by acute or protracted exposure^(5)^. The previously recommended thresholds were 2–10 Gy for single brief exposures and above 8 Gy for protracted exposures^(6)^. The ICRP 2012 states that ‘the new recommended equivalent dose limit for occupational exposure of the lens of the eye is based on prevention of radiogenic cataracts’. The new ICRP recommendation has been adopted by the European Union (EU) in the Directive 2013/59/EURATOM^(7)^ and by the International Atomic Energy Agency (IAEA), in the new international basic safety standards publication^(8)^. Subsequently, many states, Finland included, have implemented the equivalent dose limits for the lens of the eye from the new basic safety standards into national legislation (Finnish Radiation Act 859/2018, 2018, Finnish Government Decree on Ionising Radiation 1034/2018, 2018). According to ICRU 51^(9)^, operational quantities (personal dose equivalents) can be used as surrogates for protection quantities. Therefore, in the case of the lens of the eye, H_p_(3) (i.e. personal dose equivalent to the lens of the eye) can be used to estimate the protection quantity equivalent dose to the lens of the eye.

In nuclear medicine, the work involves tasks with close contact to a wide range of radionuclides that cause exposure to gamma, beta and alpha radiation. Of these, gamma radiation and sufficiently high energy beta radiation may penetrate to the eye lens, and potentially induce high doses and radiation effects to the workers. The recent epidemiological evidence demonstrating the radiosensitive characteristics of the lens of the eye and the new legislation for annual limits for the lens have led to increased awareness of occupational eye lens exposure, including the exposure among nuclear medicine workers. Dabin et al.^(10)^ monitored H_p_(3) for 45 nuclear medicine staff members and estimated the annual dose to range between 0.6 and 9.3 mSv. Other investigations have reported annual H_p_(3) of less than 2 mSv for workers at a PET radiopharmaceutical facility^(11)^ and also a PET/CT centre^(12)^. One of the objectives of this study was to measure H_p_(3) among workers in two nuclear medicine units in Finland. Further, instead of measuring H_p_(3), the possibility to estimate H_p_(3) using other operational quantities, particularly H_p_(10), was investigated. Also, applying the measured H_p_(3)/H_p_(10) ratios and records from dose register, annual H_p_(3) was estimated for technicians who had been working in nuclear medicine units in Finland during a period of 10 years. To our knowledge, the H_p_(3)/H_p_(10) ratio approach utilising dose register H_p_(10) data for estimation of H_p_(3) levels for nuclear medicine workers has not been demonstrated previously.

## MATERIALS AND METHODS

### TLD calibration

Thermoluminescent detectors (MCP-N, Radcard, Poland) inserted into EYE-D™ dosemeters (Radcard) were used for measuring H_p_(3). The decision was made to perform calibration with ^137^Cs, taking into consideration the range of photon energy of the radionuclides used in nuclear medicine and that these rarely emit beta particles or positrons with a range larger than 3 mm in polyamide, i.e. the capsule material in the EYE-D dosemeter. The detectors were calibrated against ^137^Cs at the secondary standard dosimetry laboratory of Radiation and Nuclear Safety Authority (STUK). All detector readings were performed with a TOLEDO 654 reader (Vinten Instruments Limited, UK). A linear response for 10 doses from 100 μSv to up to 20 mSv was achieved using slab phantom, and the results were verified using the PMMA 20 cm x 20 cm cylinder phantom^(13)^. A blinded performance test was conducted showing that the MCP-N detector in the eye dosemeter was able to detect doses below 40 μSv.

Energy response of the eye dosemeters was tested with the slab phantom using 10 x-ray ISO narrow qualities (ISON 25 to ISON 250; ISO 4037-1, 1996) with mean energy ranging from 20 to 208 keV and showing a ratio of relative response (the response of ISO N qualities with respect to ^137^Cs) between 0.8 and 1.2 resembling to a large extent the response with the cylinder phantom performed by Bilski et al.^(13)^. Using the cylinder phantom, the energy response was repeated with ISO N80, N120, N250 and ^137^Cs, the results showing that the relative response for these energies was within 3% of the response for the slab phantom. Since the dosemeters were irradiated from different directions in various work tasks at the nuclear medicine units, the angle of incidence was tested for ISO N80, N250 and ^137^Cs using the cylinder phantom. The tested angles were 45° from both front and reverse side of the EYE-D capsule and, also 90°, i.e. from the side of the capsule, and compared to reference direction 0°. The results showed that all angles were well in line with the 0° response, except for the 90° where an overresponse for the tested energies was observed. For 662 keV gamma radiation, the influence was of the order of 5% and for 65 keV about 25% (data not shown).

### Study participants and measurements at nuclear medicine units

Clearance for conducting the investigation was obtained from the respective hospital administrations. Volunteers from two nuclear medicine units participated in the study. Since person identification was necessary for the retrieval of individual dose register data (including dose values below national recording threshold, i.e. 0.1 mSv for H_p_(10)), informed consent was requested from the study participants. Individual data were coded, and the data from dose register and in the study were processed anonymously.

H_p_(3) was measured among 7 and 9 technicians at nuclear medicine units at two university hospitals (UH1 and UH2), respectively. The dosemeter was attached to the arm of personal eyewear in most cases except for two persons who wore the dosemeter in a head band at UH2. The participants were instructed to place the dosemeter as near eye level as possible, whereas no guidance was given about which side of the head the EYE-D dosemeter should be located. This decision was made since there was no predominant angle or orientation of the handled sources with respect to the eye dosemeter in any of the work tasks. Measurement periods varied between 8 and 20 working days and were synchronised with the periods of the official staff dosemeters for measuring personal dose equivalent H_p_(10), i.e. each person wore one eye dosemeter and one whole body dosemeter during the measurement period. The seven participants at UH1 wore eye dosemeters during several measurement periods resulting in 23 dose values. At UH2, only one measurement period was conducted for each participant. Out of 32 measurement periods, two were omitted due to one misplacement of dosemeter and one broken dosemeter during the measurement period. Background dose was monitored for both H_p_(3) and H_p_(10) by placing dosemeters in rest areas/locker rooms within the premises of the nuclear medicine units. When not in use, all measurement dosemeters were stored together with those dosemeters allocated for background monitoring.

Work tasks included the following task entities: operation of PET-CT and gamma camera/SPECT-CT, preparation of radiopharmaceuticals, patient preparation and care. The staff rotated in various work tasks. Among UH1 participants, the aim was to recruit technicians working especially with PET tasks in order to potentially identify the highest doses. The work tasks included handling of the following nuclides: mainly ^18^F, ^68^Ga and ^99m^Tc and to a lesser extent ^123^I, ^111^In, ^68^Ge, ^22^Na and ^57^Co. In 2017 at UH1, a total amount of 6550 patient studies were conducted, excluding radionuclide therapy treatments handled by doctors. Of these, 600 were ^68^Ga-Dotanoc or PSMA studies and 2050 ^18^F-FDG-PET studies.

At UH2, the work tasks included handling of the following nuclides: mainly ^99m^Tc, ^18^F and ^57^Co and to a lesser extent ^68^Ga, ^131^I and ^123^I. The total number of patient studies in 2017 was 2640. Of these, 57 were ^68^Ga-PSMA studies and 817 ^18^F-FDG-PET studies. At neither nuclear medicine department did the workers use personal shields such as thyroid shield, lead apron shield or lead glasses while working on PET and gamma camera/SPECT-CT related tasks. At both departments, an automatic injector was used for ^18^F-FDG injections. The participating personnel in both nuclear medicine units were asked to keep record on the approximate time spent at each work entity by using a specific form.

### Dosimetry

Estimation of H_p_(3) received during one measurement period was achieved by multiplying the background-corrected TLD reading with the sensitivity coefficient obtained from ^137^Cs calibration. The annual dose for each worker was estimated by firstly calculating an average daily dose based on the measured values and length of measurement period and thereafter multiplying this with an estimated number of working days (220) during a year.

Whole body H_p_(10) for the workers was measured using TLD-100 dosemeter. The dosemeters and the measurement services were provided by approved dosimetry service Doseco (Jyväskylä, Finland), and the dose data were obtained from the national dose register at STUK.

The uncertainty estimation included dosemeter repeatability, individual sensitivity of dosemeter (batch homogeneity), energy and angle responses as well as dose calibration. Expanded uncertainty (*k* = 2) of H_p_(3) was estimated as ca. 18%, and for H_p_(10), this was estimated as 24% (Doseco).

### Estimation of H_p_(3) using H_p_(10) obtained from national dose register

To study the exposure of technicians working in nuclear medicine on national scale, H_p_(3) was estimated from annual H_p_(10) data gathered from the national dose register. The ratio H_p_(3)/H_p_(10) = 0.7 estimated based on the TLD measurements was applied to calculate H_p_(3) from the dose register data.

The data covered all annual H_p_(10) entries for nuclear medicine technicians between the years 2009 and 2018, a total of 2813 entries. Since a 0.1 mSv recording threshold for one measurement period of H_p_(10) is applied for the entries in the dose register, the annual H_p_(10) for category B workers was reported as zero or slightly above zero. Thus, data analysis was limited to the dose records of category A workers, with 1721 register entries.

## RESULTS AND DISCUSSION

### Individual H_p_(3) and H_p_(10) measurements

**Figure 1:**
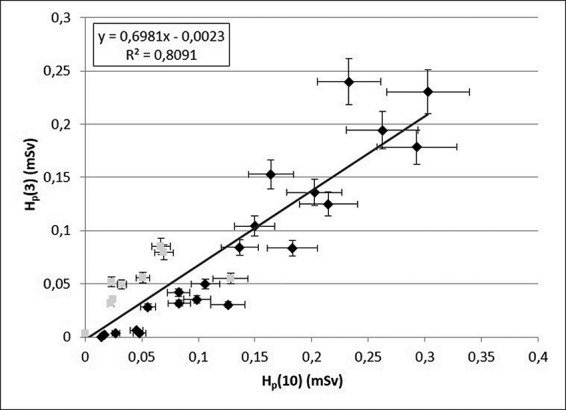
Correlation of H_p_(3) with respect to H_p_(10). Each plotted value represents one dose measurement covering 8–20 days of work at nuclear medicine units. Black symbols stand for UH1 and grey symbols for UH2. Error bars represent measurement uncertainties (1 σ).

Individual H_p_(3) measured with the EYE-D dosemeters and H_p_(10) from personal TLD-100 dosemeters are plotted in Figure 1. The doses for single measurement periods ranged from less than 10 to 250 μSv for H_p_(3) and to 300 μSv for H_p_(10). The variation in the measured doses reflects the range of work tasks among participants and is also dependent on differences in the length of measurement period. The doses acquired at UH2 nuclear medicine unit are clearly lower than doses at the UH1 unit. Table 1 shows that the content of work tasks between the participating technicians in the two nuclear medicine units was indeed dissimilar, the portion of recorded working time allocated for PET related tasks being on average 57% for UH1 technicians, whereas at UH2, the technicians used on average 12% of their working time for PET operations, excluding PET-TT imaging of patients, including ^18^F-FDG automatic dispenser loading, patient dose administrating, injections and ^68^Ga labeling and manual injections. The estimated annual H_p_(10) and H_p_(3) and the number of measurement periods for each technician are also presented in Table 1. The annual doses were estimated with the underlying assumption that the work tasks and conditions would remain unchanged from what they were during the measurement period. The highest annual H_p_(3) was 3.9 mSv for a technician who wore the dosemeter only for a single measuring period and whose work tasks consisted almost entirely of PET work.

**Table 1:** Number of measurement periods, estimated annual H_p_(3) and H_p_(10) ± measurement uncertainty (1 σ) and approximated percentage of PET work for each nuclear medicine worker

Nuclear medicine unit	Worker nr.	Nr. of measurement periods	H_p_(10) (mSv)	H_p_(3) (mSv)	Portion of PET-related work tasks (%)
	1	3	1.3 ± 0.2	0.4 ± 0.04	22
	2	3	2.9 ± 0.3	2.2 ± 0.2	44
	3	4	3.2 ± 0.4	1.8 ± 0.2	99
UH1	4	4	0.4 ± 0.05	0.05 ± 0.004	6
	5	1	5.2 ± 0.6	3.9 ± 0.3	100
	6	4	1.9 ± 0.2	1.2 ± 0.1	91
	7	2	2.8 ± 0.3	2.1 ± 0.2	36
		∑ 21	Mean 2.5 ± 0.3	1.7 ± 0.1	57
					
	1	1	0.6 ± 0.1	0.6 ± 0.1	11
	2	1	1.4 ± 0.2	0.6 ± 0.1	2
	3	1	0.5 ± 0.1	0.7 ± 0.1	11
UH2	4	1	0.8 ± 0.1	1.0 ± 0.1	14
	5	1	0.4 ± 0.04	0.8 ± 0.1	37
	6	1	0.3 ± 0.04	0.4 ± 0.04	0
	7	1	0.4 ± 0.05	0.6 ± 0.1	8
	8	1	0.8 ± 0.1	1.0 ± 0.1	23
	9	1	0.0	0.05 ± 0.004	0
		∑ 9	Mean 0.6 ± 0.1	0.6 ± 0.1	12
Total		30	Mean 1.4 ± 0.2	1.1 ± 0.1	

The relatively low annual H_p_(3) observed in this study implies that the equivalent dose to the lens of the eye among technicians working at two nuclear medicine units in Finland remain far below the annual dose limits enforced in the new radiation legislation. Similar results have been obtained in other studies on nuclear medicine departments, showing estimated maximum annual H_p_(3) of 3.7 mSv^(14)^, about 2.5 mSv^(15)^, 9.3 mSv^(10)^, 4.5 mSv^(16)^ and 8 mSv^(17)^. Considering the ICRP recommendations of dose limits to the lens of the eye, these investigations imply that the risk for radiogenic cataracts is comparatively low among technicians conducting routine work tasks at nuclear medicine departments.

### H_p_(3)/H_p_(10) ratio

**Figure 2:**
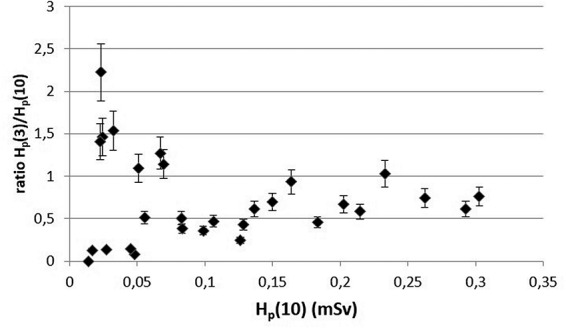
Ratio H_p_(3)/H_p_(10) with respect to H_p_(10). Error bars represent combined measurement uncertainty (1 σ) from both dose qualities.

Figure 1 illustrates the relationship of H_p_(3) with H_p_(10) and demonstrates a good H_p_(3)/H_p_(10) correlation (linear regression equation slope of 0.70, Pearson’s coefficient *r* = 0.90, *p* < 0.001). Excluding two measurements in which the doses were practically zero, the individual H_p_(3)/H_p_(10) ratios varied between 0.1 and 2.3. The vast majority of the individual H_p_(3)/H_p_(10) ratios remained well under unity, as demonstrated in Figure 2. It also shows that all ratios above unity could be found for H_p_(10) below 0.1 mSv. This distinction was also reflected in the ratios among the two nuclear medicine units: the ratio for UH1 ranged between 0.1 and 1.0, whereas at UH2 where the individual measurements showed low doses in general, the ratios were between 0.4 and 2.2. Overall, there was a clear difference in homogeneity of individual H_p_(3)/H_p_(10) ratios in the group belonging to H_p_(10) below 0.1 mSv in comparison to the group above 0.1 mSv (*σ*^2^ of 0.43 and 0.04, respectively), indicating that the estimation of H_p_(3) is more reliable at higher H_p_(10) values. Our results are in line with findings obtained in other studies conducted on workers at nuclear medicine departments. Dabin et al.^(10)^ found a clear correlation between H_p_(10) measured at chest level and H_p_(3) among 45 staff members, although the linear correlation was relatively poor (Pearson’s coefficient *r* = 0.62). In other studies, the ratios were reported near unity for five technicians and three nurses^(15)^, whereas an average H_p_(3)/H_p_(10) ratio of 0.55 was calculated based on 3-month measurements among 19 workers^(14)^. All these investigations give strong implications to the use of H_p_(10) as an indicator of H_p_(3) levels for nuclear medicine workers. Further support comes from a study by Kopec et al.^(18)^ who found ratios of H_p_(3)/H_p_(10) between 0.7 and 1.1 among technical staff and nurses at one scintigraphy and two PET-CT departments. They also demonstrated the importance of appropriate placement of the eye dosemeter for achieving correct measurements. Walsh et al.^(12)^ found H_p_(3) values measured by eye dosemeters to be within 50% of the measured numeric H_p_(10) values. The above studies applied the MCP-N detector in the eye dosemeter. Measurements performed with the TLD-100 chip showed up to 200% higher H_p_(3) values in comparison to dose to the thorax^(19)^. Several factors may affect the magnitude of the H_p_(3)/H_p_(10) ratio, one of them being the height of the person. In interventional radiology, the ratio was shown to be affected by the height of the radiologist so that the ratio decreased with increasing height^(20)^.

### Angular and energy effects

In most cases in the present study, the EYE-D dosemeters were worn attached to the personal eyeglasses so that the black polyamide capsule pointed outwards from the side of the head. The measurements would ideally be performed with eye dosemeters located on the forehead as close to the eye as possible, e.g. placed on a head band. Thus, the eyeglass attachment can be regarded as a limitation of the study. While turning the head in relation to the irradiation source, irradiation from the front of the EYE-D capsule, from the side and also from the back was enabled. Gamma radiation emitted by typical nuclear medicine radionuclides covers the energy range of about 0.1–0.66 MeV. Angular tests covering this energy range indicated that attaching the dosemeter to eyeglasses so that they are irradiated from the side induces larger uncertainties to dose estimate than irradiation to the front of the EYE-D. However, the overresponse lies well within the limits for angular response of 0.67–1.67^(21)^, although these limits are only given for maximum of 60° angle. The data also indicate that the overresponse for irradiation from the side increases with decreasing energy. Although the attachment of the dosemeter was not ideal, the overall assessment is that irrespective of the angle of the eye dosemeter with respect to source, and with energies covering typical nuclear medicine nuclides, a relatively uniform response in the EYE-D dosimetry is induced. Attaching the dosemeter to personal eyewear naturally rules out the possibility to monitor persons not wearing glasses. It should be also kept in mind that different eyewear models and materials may influence the measurement.

Many of the radionuclides handled at the nuclear medicine departments are beta emitters, and electrons and positrons with energies of less than ca. 700 keV are absorbed by the 3 mm polyamide capsule on the EYE-D as illustrated by Szermerski et al.^(22)^. The study pointed out that betas from ^131^I and positrons from ^18^F are fully attenuated, whereas electrons and positrons with higher energies, e.g. from ^90^Y and ^68^Ga, respectively, are penetrating the polyamide layer and thus contributing to the dose. Also, it was shown that the polyamide layer and the detector together form a 4.6 mm thick layer of tissue-equivalent material, i.e. much thicker than the 3 mm in the definition of H_p_(3). Szumska et al.^(23)^ investigated the MCP-N detector for beta emitters ^32^P, ^42^K and ^90^Sr/^90^Y and showed that there is an increasing response for the detector with increasing energy of beta emitters.

Bruchmann et al.^(24)^ have studied the influence of protective eyewear on H_p_(3) by exposure of head phantoms with radionuclides commonly used in nuclear medicine and that were emitting gamma and/or beta/positron radiation. They found that the attenuation effect for H_p_(3) of both ordinary laboratory glasses and leaded protective glasses was highest at exposure to ^90^Y (factor < 0.1). The attenuation factor for lead glasses was approximately 0.3, 0.9, 0.5 and 0.8 for ^99m^Tc, ^18^F, ^68^Ga and ^131^I, respectively, whereas the laboratory glasses were much less protective. In interventional radiology where lead glasses are often used by the interventionist, the positioning of the eye dosemeter has a substantial impact on the ratio between the measured H_p_(3) and actual eye lens equivalent dose^(25)^. No leaded eyewear was used in our study and the energy spectra and the homogeneity of the radiation fields within radiology diverge considerably in comparison to those encountered in nuclear medicine. However, it should be kept in mind that although the protection quantity equivalent dose can be replaced by H_p_(3) for monitoring purposes, there may be differences in the magnitude of these two quantities.

**Figure 3:**
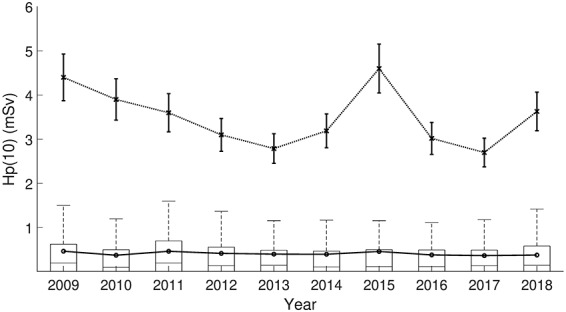
Statistics of measured H_p_(10) values from the national dose register between 2009 and 2018. Boxes: upper edge = 75th percentile, centre line = median. Whiskers = 1.5 interquartile ranges. Solid line: average, dotted line with error bars = maximum H_p_(10) values ± measurement uncertainty (1 σ).

**Figure 4:**
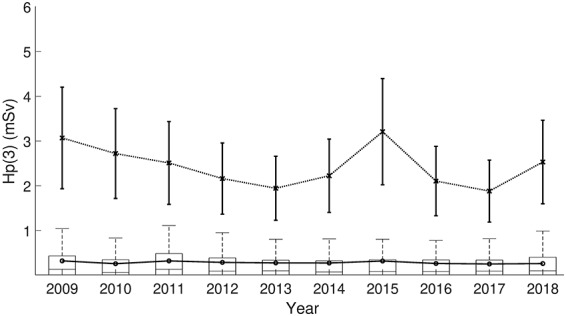
Statistics of H_p_(3) estimates obtained by applying the regression model from Figure 1 to H_p_(10) records from national dose register. Boxes: upper edge = 75th percentile, centre line = median. Whiskers = 1.5 interquartile ranges. Solid line = average, dotted line with error bars = calculated maximum H_p_(3) values and 95% prediction interval for the regression model.

### H_p_(3) calculated from dose register data

Based on national dose register data for category A technicians working in nuclear medicine, Figures 3 and 4 show respective boxplots of annual H_p_(10) and the ratio-derived H_p_(3) values between 2009 and 2018. During the 10 year time span, the annual H_p_(10) on national level was on average 0.41 mSv and the mean of the derived H_p_(3) 0.28 mSv, also displayed in Figures 3 and 4. The figures also show maximum values for each year and during the 10-year period, the highest H_p_(10) was 4.6 ± 0.6 mSv and the derived-H_p_(3) 3.2 ± 1.2 mSv. The effect of possible non-compliance to wearing personal dosemeters was not accounted for, which may potentially underestimate the results derived based on the dose register data. Compared to the average annual H_p_(3) doses in the overall national cohort, much higher average annual H_p_(3) dose (1.1 mSv, Table 1) was measured among the 16 technicians participated in the study. This was due to recruitment of technicians working particularly with PET-related tasks where high doses are potentially identified. The conclusion of the above is that for nuclear medicine workers, the annual equivalent dose levels would very unlikely reach the allowed dose limit.

## CONCLUSIONS

The results of this study, performed for technicians at two nuclear medicine departments, indicate that H_p_(3), considered as a surrogate for the protection quantity equivalent dose to the lens of the eye, does not rise anywhere near the mean value of 20 mSv over a five-year period. Good correlation between the two operational quantities suggests that the ratio H_p_(3)/H_p_(10) is a good candidate for estimation of H_p_(3) from H_p_(10) among nuclear medicine workers. Based on 10 years of H_p_(10) data from nuclear medicine technicians in Finland, the ratio-estimated annual H_p_(3) doses were shown to stay well below the 20 mSv annual limit also long-term. Taking into account the potential role of H_p_(10) for determining dose to the lens of the eye, and the relatively low dose level observed in this study, there is no clear indication to recommend routine H_p_(3) measurement among technicians working in nuclear medicine imaging departments.
